# Development and validation of a 25-Gene Panel urine test for prostate cancer diagnosis and potential treatment follow-up

**DOI:** 10.1186/s12916-020-01834-0

**Published:** 2020-12-01

**Authors:** Heather Johnson, Jinan Guo, Xuhui Zhang, Heqiu Zhang, Athanasios Simoulis, Alan H. B. Wu, Taolin Xia, Fei Li, Wanlong Tan, Allan Johnson, Nishtman Dizeyi, Per-Anders Abrahamsson, Lukas Kenner, Xiaoyan Feng, Chang Zou, Kefeng Xiao, Jenny L. Persson, Lingwu Chen

**Affiliations:** 1 Olympia Diagnostics, Inc., Sunnyvale, CA USA; 2 Department of Urology, The Second Clinical Medical College of Jinan University, Shenzhen People’s Hospital, Shenzhen Urology Minimally Invasive Engineering Centre, Shenzhen, China; 3 Shenzhen Public Service Platform on Tumor Precision Medicine and Molecular Diagnosis, Clinical Medical Research Centre, The Second Clinical College of Jinan University, Shenzhen People’s Hospital, Shenzhen, China; 4 Department of Bio-diagnosis, Institute of Basic Medical Sciences, Beijing, China; 5 Department of Clinical Pathology and Cytology, Skåne University Hospital, Malmö, Sweden; 6 Clinical Laboratories, San Francisco General Hospital, San Francisco, CA USA; 7 Department of Urology, Foshan First People’s Hospital, Foshan, China; 8 Department of Urology, Nanfang Hospital, Southern Medical University, Guangzhou, China; 9 Kinetic Reality, Santa Clara, CA USA; 10 Department of Translational Medicine, Lund University, Clinical Research Centre, Malmö, Sweden; 11 Department of Experimental Pathology, Medical University Vienna & Unit of Laboratory Animal Pathology, University of Veterinary Medicine, Vienna, Austria; 12 Department of Molecular Biology, Umeå University, 901 87 Umeå, Sweden; 13 Division of Experimental Cancer Research, Department of Translational Medicine, Lund University, 205 02 Malmö, Sweden; 14 Department of Biomedical Sciences, Malmö University, Malmö, Sweden; 15 Department of Urology, The First Affiliated Hospital of Sun Yat-Sen University, Guangzhou, 510080 Guangdong China

**Keywords:** Prostate cancer, Prostate cancer diagnosis, Clinically significant prostate cancer, Prostate cancer treatment follow-up, Gene Panel, Urine test

## Abstract

**Background:**

Heterogeneity of prostate cancer (PCa) contributes to inaccurate cancer screening and diagnosis, unnecessary biopsies, and overtreatment. We intended to develop non-invasive urine tests for accurate PCa diagnosis to avoid unnecessary biopsies.

**Methods:**

Using a machine learning program, we identified a 25-Gene Panel classifier for distinguishing PCa and benign prostate. A non-invasive test using pre-biopsy urine samples collected without digital rectal examination (DRE) was used to measure gene expression of the panel using cDNA preamplification followed by real-time qRT-PCR. The 25-Gene Panel urine test was validated in independent multi-center retrospective and prospective studies. The diagnostic performance of the test was assessed against the pathological diagnosis from biopsy by discriminant analysis. Uni- and multivariate logistic regression analysis was performed to assess its diagnostic improvement over PSA and risk factors. In addition, the 25-Gene Panel urine test was used to identify clinically significant PCa. Furthermore, the 25-Gene Panel urine test was assessed in a subset of patients to examine if cancer was detected after prostatectomy.

**Results:**

The 25-Gene Panel urine test accurately detected cancer and benign prostate with AUC of 0.946 (95% CI 0.963–0.929) in the retrospective cohort (*n* = 614), AUC of 0.901 (0.929–0.873) in the prospective cohort (*n* = 396), and AUC of 0.936 (0.956–0.916) in the large combination cohort (*n* = 1010). It greatly improved diagnostic accuracy over PSA and risk factors (*p* < 0.0001). When it was combined with PSA, the AUC increased to 0.961 (0.980–0.942). Importantly, the 25-Gene Panel urine test was able to accurately identify clinically significant and insignificant PCa with AUC of 0.928 (95% CI 0.947–0.909) in the combination cohort (*n* = 727). In addition, it was able to show the absence of cancer after prostatectomy with high accuracy.

**Conclusions:**

The 25-Gene Panel urine test is the first highly accurate and non-invasive liquid biopsy method without DRE for PCa diagnosis. In clinical practice, it may be used for identifying patients in need of biopsy for cancer diagnosis and patients with clinically significant cancer for immediate treatment, and potentially assisting cancer treatment follow-up.

**Supplementary Information:**

The online version contains supplementary material available at 10.1186/s12916-020-01834-0.

## Background

Prostate cancer (PCa) is the second most prevalent cancer and a leading cause of cancer-related death [1]. Needle biopsy is a standard method for PCa diagnosis, yet it is invasive and associated with complications and missing lesions [2]. Prostate-specific antigen (PSA) is a widely used PCa screening test, yet with moderate sensitivity and very low specificity (< 30%), resulting in > 70% false positive rate and many unnecessary biopsies [2]. Although tests using PSA isoforms/analogs have been developed, their improvement on accuracy is limited [2, 3]. For clinically meaningful PCa diagnosis, it is important to identify patients with clinically significant cancer. Although the new tools such as magnetic resonance imaging (MRI) and multiparametric MRI targeted biopsy have been used to identify patients with clinically significant PCa, these methods have limited accuracy [4–6].

During tumorigenesis, PCa cells are exfoliated from the prostate and released into the urine [7], making urine a readily available source to detect prostate-specific biomarkers for diagnosis and prognosis. Although many urine biomarkers have been identified and used individually or in combination for diagnosis, none of them has sensitivity and specificity both above 90% and AUC above 0.9. Most studies tested in < 300 samples. All of them use urine collected after digital rectal examination (DRE), which is invasive and uncomfortable for patients [2, 6, 8–12]. In addition, with very low specificity of the PSA test for cancer diagnosis and limitation of imaging technologies to identify residual cancer lesions after treatment, no accurate test is available to assess efficacy and outcome of PCa treatment such as prostatectomy. Yet it is crucial to accurately measure treatment outcome to assist treatment decision-making, such as assessing if residual cancer lesion remains after prostatectomy to determine the necessity of subsequent treatment, leading to improved cancer treatment and reduced mortality [13, 14]. Therefore, it is of great clinical significance to develop better tests for these unmet medical needs.

PCa is a cancer with a high degree of heterogeneity. Many gene alterations contribute to cancer tumorigenesis, progression, recurrence, and metastasis [15]. Thus, it is necessary to combine multiple biomarkers involved in these processes.

We therefore developed a novel 25-Gene Panel urine test for PCa diagnosis and potential treatment follow-up. We showed that the test was robust with high accuracy in two independent, multi-center studies.

## Methods

### Retrospective and prospective studies

A multi-center retrospective study was approved by the Institutional Review Board (IRB) of San Francisco General Hospital (San Francisco, USA) (IRB # 15-15816) to collect and test archived urine sediments to identify and validate urine biomarkers for PCa diagnosis. The prospectively designed, retrospectively collected pre-biopsy urine samples were randomly picked from sample archives at Cooperative Human Tissue Network (CHTN) Southern Division (patients in the USA) and Indivumed GmbH (patients in Germany). The urine samples were collected from patients with elevated PSA scheduled for biopsy for cancer diagnosis from July 2004 to November 2014 with prior ethical approval and patient consent for future studies. A multi-center prospective study for urine biomarkers was approved by IRB of Shenzhen People’s Hospital (Shenzhen, China) (Study Number P2014-006) to collect pre-biopsy fresh urine samples from patients treated at seven hospitals collaborated in the study with patient consent, including Shenzhen People’s Hospital, The First Affiliated Hospital of Sun Yat-Sen University, Peking University First Hospital, Foshan First People’s Hospital, Nanfang Hospital at Southern Medical University, Peking University Shenzhen Hospital, and The Second People’s Hospital of Shenzhen. The urine samples were collected consecutively from patients with elevated PSA scheduled for biopsy in the participating hospitals. Both studies used the same patient inclusion criteria of age at 18–85, with histopathological diagnosis of PCa, BPH, or prostatitis from biopsy, and without treatment of PCa drugs or 5-alpha reductase inhibitors prior to urine collection. The exclusion criteria included having prostatectomy or treatment with PCa drugs or 5-alpha reductase inhibitors before urine collection. In addition, ten patients undergoing prostatectomy were recruited to collect urine samples several days before and after surgery. The pathological diagnosis of PCa in both retrospective and prospective studies was performed by using standard needle biopsy with consistent procedures. The pathological diagnosis of clinically significant or insignificant PCa was defined based on PCa risk stratification guidelines from the National Comprehensive Cancer Network (NCCN) with modifications. The clinically significant PCa patients were classified as meeting any of the following criteria: Gleason score > 7, Gleason score 4 + 3 = 7, cancer staging ≥ T3, PSA > 20 ng/mL at diagnosis, biochemical recurrence after prostatectomy during the follow-up period, or cancer metastasis at diagnosis or during the follow-up period. The rest of the patients were classified as clinically insignificant PCa. All samples were de-identified and coded with patient numbers to protect patient privacy following the Health Insurance Portability and Accountability Act guidelines. Urine samples from 665 patients were received with 51 excluded in the retrospective cohort and urine samples from 411 patients were received with 15 excluded in the prospective cohort respectively, due to the lack of pathology report, diagnosis uncertainty, or low/no gene expression detected.

### Urine processing and quantification of gene expression

For the retrospective study, 10–15 mL urine samples were collected without digital rectal examination (DRE) and the urine pellet was flash-frozen and stored at − 80 °C. For the prospective study, 15–45 mL urine without DRE was collected in the presence of 5 mL DNA/RNA preservative AssayAssure (Thermo Fisher Scientific, Waltham, MA, USA) or U-Preserve (Hao Rui Jia Biotech Ltd., Beijing, China), stored at 4 °C, and processed within 7 days. The urine pellet obtained after centrifugation at 1000×*g* for 10 min was washed with phosphate-buffered saline followed by a second centrifugation at 1000×*g* for 10 min. The cell pellet was processed for RNA purification or immediately frozen on dry ice and stored at − 80 °C. A detailed procedure of gene expression quantification is listed in Additional file 1: Methods.

### Prostate tissue specimen cohort

The GSE17951 prostate tissue specimen cohort includes quantitative mRNA expression data of PCa and benign prostate specimens obtained from Affymetrix U133Plus2 array [16, 17]. The PCa tissues (*n* = 56) in the cohort were collected from patient biopsy specimens, and the benign prostate tissues (*n* = 98) were obtained from prostate autopsy specimens of patients with benign disease. The gene expression levels of the 25 genes in the panel were obtained from the database and normalized with beta-actin expression level.

### Data analysis and algorithm for cancer diagnosis

All data analysis and diagnosis by the 25-Gene Panel were performed blindly without prior knowledge of patient information. The gene expression data was downloaded and first analyzed with ABI Quantstudio 6 software (Thermo Fisher Scientific, Waltham, MA, USA). The mRNA expression level of the housekeeping gene beta-actin was measured in each urine sample and used for gene expression normalization to control variation of cDNA quantity in the urine samples. The cycle threshold (Ct) value of each gene in the panel was divided by the Ct value of the beta-actin and then multiplied by 1000 as the normalized gene expression value (CtS = Ct (sample)/Ct (actin) × 1000). For each gene, the average Ct value from triplicate PCR was used. For the diagnosis of cancer by the 25-Gene Panel, the relative Ct (CtS) values of the 25 genes in the panel were used to generate a classification score (diagnostic *D* score).

For cancer diagnosis in both retrospective and prospective cohorts, each sample was diagnosed using the Diagnosis Algorithm as shown below:

$$ {C}_{\mathrm{PCa}}={A}_{\mathrm{PCa}}+{\mathrm{CtS}}_1\ast {X}_1+{\mathrm{CtS}}_2\ast {X}_{2\dots }+{\mathrm{CtS}}_{25}\ast {X}_{25}+{\mathrm{CtS}}_1\ast {\mathrm{CtS}}_1\ast {X}_{1\ast 1}+{\mathrm{CtS}}_1\ast {\mathrm{CtS}}_2\ast {X}_{1\ast 2\dots }+{\mathrm{CtS}}_1\ast {\mathrm{CtS}}_{25}\ast {X}_{1\ast 25}+{\mathrm{CtS}}_2\ast {\mathrm{CtS}}_2\ast {X}_{2\ast 2\dots }+{\mathrm{CtS}}_2\ast {\mathrm{CtS}}_{25}\ast {X}_{2\ast 25\dots }+{\mathrm{CtS}}_{25}\ast {\mathrm{CtS}}_{25}\ast {X}_{25\ast 25} $$

$$ {C}_{\mathrm{Non}}={B}_{\mathrm{Non}}+{\mathrm{CtS}}_1\ast {Y}_1+{\mathrm{CtS}}_2\ast {Y}_{2\dots }+{\mathrm{CtS}}_{25}\ast {Y}_{25}+{\mathrm{CtS}}_1\ast {\mathrm{CtS}}_1\ast {Y}_{1\ast 1}+{\mathrm{CtS}}_1\ast {\mathrm{CtS}}_2\ast {Y}_{1\ast 2\dots }+{\mathrm{CtS}}_1\ast {\mathrm{CtS}}_{25}\ast {Y}_{1\ast 25}+{\mathrm{CtS}}_2\ast {\mathrm{CtS}}_2\ast {Y}_{2\ast 2\dots }+{\mathrm{CtS}}_2\ast {\mathrm{CtS}}_{25}\ast {Y}_{2\ast 25\dots }+{\mathrm{CtS}}_{25}\ast {\mathrm{CtS}}_{25}\ast {Y}_{25\ast 25} $$

Diagnostic *D* score = *C*_PCa_ − *C*_Non_

Whereas *A*_PCa_ is the PCa constant, *B*_Non_ is the non-PCa constant, CtS_1_ through CtS_25_ are CtS values of gene 1 through gene 25, *X*_1_ through *X*_25_ are PCa regression coefficients of gene 1 through gene 25, *X*_1*1_ through *X*_25*25_ are gene 1 and gene 1 cross PCa regression coefficients through gene 25 and gene 25 cross PCa regression coefficients, *Y*_1_ through *Y*_25_ are non-PCa regression coefficients of gene 1 through gene 25, and *Y*_1*1_ through *Y*_25*25_ are gene 1 and gene 1 cross non-PCa regression coefficients through gene 25 and gene 25 cross non-PCa regression coefficients. The sample was diagnosed to be PCa when the diagnostic *D* score was > 0, whereas the sample was diagnosed to be benign prostate (non-PCa) when the diagnostic *D* score was ≤ 0.

### Statistical analysis

To generate an algorithm for diagnosing urine samples as PCa or benign prostate (Diagnosis Algorithm), discriminant analysis was performed to test the association between pathological diagnosis and CtS values of the 25 genes in the panel using a statistical software program XLSTAT (Addinsoft, Paris, France). The diagnosis of all the samples by the algorithm was compared to their pathological diagnosis to assess diagnostic performance by calculating sensitivity, specificity, positive predictive value, negative predictive value, odds ratio, and their respective 95% confidence intervals. The receiver operating characteristic curve was plotted and the area under the curve (AUC) with its 95% confidence interval was calculated. To further validate the 25-Gene Panel in the combination cohort, the leave-one-out cross-validation analysis was performed to generate regression coefficients to determine the classification of each sample by the 25-Gene Panel, which was then compared with the pathological diagnosis of each sample to calculate the diagnostic performance of cross-validation using XLSTAT. In addition, univariate and multivariate logistic regression analyses were conducted to compare the diagnostic performance of pre-biopsy PSA, pre-biopsy PSA at the cutoff value of 4 ng/mL, patient age, PCa family history, the 25-Gene Panel urine test, and their combinations.

## Results

### Non-invasive urine test

Current urine tests for PCa diagnosis and prognosis rely on DRE before urine collection to enrich prostate cells in the urine, yet the procedure is uncomfortable and invasive for patients and requires a physician to perform. To develop a non-invasive urine test to measure gene expression of biomarkers, we employed a modified method of cDNA preamplification before real-time qRT-PCR [18] and showed that it improved quantification of gene expression in urine collected without DRE that contained fewer prostate cells. We detected mRNA expression of the genes with significantly increased sensitivity by ~ 10 Ct units without changing the relative gene expression values (ΔCt) (Additional file 2: Table S1). The ΔCt values were similar in the urine samples collected from the same patients with and without DRE (Additional file 2: Table S2), the urine with and without DRE had similar diagnostic *D* score, and the diagnosis of the urine with or without DRE was the same (Table 1). With the help of DNA/RNA preservative, urine can be collected without DRE or physician’s involvement and stored or shipped at room temperature within a week. Our data demonstrated that the new method developed in the study is robust and can be used to quantify biomarker gene expression in urine samples without DRE, making it a valid and much improved liquid biopsy method in clinical practice.

**Table 1:** Diagnosis of urine samples collected with and without DRE

	***D*** score-DRE-urine	***D*** score-DRE+urine	SD	SD/mean (%)	Diagnosis-DRE-urine	Diagnosis-DRE+urine
Patient 1	30.7	31.9	0.8	2.5	PCa	PCa
Patient 2	30.4	30.1	0.3	0.8	PCa	PCa
Patient 3	30.1	30.6	0.4	1.2	PCa	PCa
Patient 4	35.0	32.9	1.5	4.3	PCa	PCa
Patient 5	30.5	29.9	0.4	1.4	PCa	PCa

*DRE* digital rectal examination, *D score-DRE-urine* diagnostic *D* score of the urine sample collected without DRE, *D score-DRE+urine* diagnostic *D* score of the urine sample collected after DRE, *diagnosis-DRE-urine* diagnosis of the urine sample collected without DRE, *diagnosis-DRE+urine* diagnosis of the urine sample collected after DRE

### Development of the 25-Gene Panel classifier

In a previous study, we identified a series of biomarker candidates involved in cell proliferation, survival, migration, tumorigenesis, cancer invasion, and metastasis with differential gene expression in PCa and benign prostate tissue specimens [19, 20]. To develop a gene panel for cancer diagnosis with high diagnostic accuracy, we used a random forest machine learning program [21, 22] combined with a discriminant analysis classification test to screen mRNA expression profiles of the biomarker candidates in PCa and benign prostate specimens in large cohorts obtained from Gene Expression Omnibus (GEO) database. The diagnosis of the specimens by various panels combining the candidate biomarkers was compared to the pathological diagnosis of the specimens to assess the diagnostic performance of the panels to distinguish PCa and benign prostate, which included diagnostic parameters of sensitivity, specificity, odds ratio, and AUC. A 25-Gene Panel consisting of *HIF1A*, *FGFR1*, *BIRC5*, *AMACR*, *CRISP3*, *FN1*, *HPN*, *MYO6*, *PSCA*, *PMP22*, *GOLM1*, *LMTK2*, *EZH2*, *GSTP1*, *PCA3*, *VEGFA*, *CST3*, *PTEN*, *PIP5K1A*, *CDK1*, *TMPRSS2*, *ANXA3*, *CCNA1*, *CCND1*, and *KLK3* was discovered to have the highest diagnostic accuracy to distinguish cancer lesions from benign prostate (Additional file 2: Table S3). We found that subtracting any one or more genes from the panel would lower the diagnostic accuracy, such as lowered sensitivity, specificity, and AUC. This showed that all genes in the panel contribute significantly to the diagnostic algorithm.

### The 25-Gene Panel urine test for cancer diagnosis

**Fig. 1:**
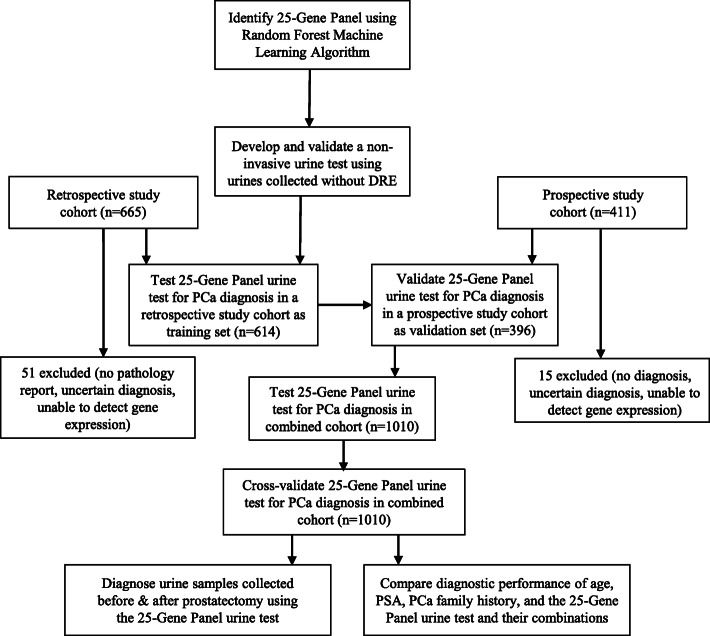
Study design

We examined if the 25-Gene Panel identified above can be used for cancer diagnosis using urine samples collected without DRE (Fig. 1). We conducted independent, multi-center retrospective and multi-center prospective studies to collect pre-biopsy urine samples and used the 25-Gene Panel as a classifier to distinguish PCa and benign prostate for diagnosis. The study population in both cohorts represents patients in real clinical practice as they are patients who underwent routine cancer diagnosis using standard PSA and biopsy in the participating hospitals. The end point of the study was to assess the diagnostic performance of the 25-Gene Panel urine test and its improvement over the known clinical parameters for PCa diagnosis. The patient characteristics and clinical parameters are illustrated based on the standard clinical practice [23] as shown in Table 2.

**Table 2:** Patient characteristics

	Retrospective cohort		Prospective cohort		Combination cohort	
	Non-PCa	PCa	Non-PCa	PCa	Non-PCa	PCa
Patients (%)	94 (15.3%)	520 (84.7%)	189 (47.7%)	207 (52.3%)	283 (28.0%)	727 (72.0%)
Mean age (year)	64 (41–84)	64 (45–78)	69 (45–86)	69 (39–88)	68 (41–86)	65 (39–88)
Patients with other cancers (%)	1 (1.1%)	4 (0.8%)	2 (1.1%)	1 (0.5%)	3 (1.1%)	5 (0.7%)
Gleason score (%)						
Group 1: ≤ 6 (≤ 3 + 3)	NA	124 (23.8%)	NA	39 (18.8%)	NA	163 (22.4%)
Group 2: 7 (3 + 4)	NA	218 (41.9%)	NA	54 (26.1%)	NA	272 (37.4%)
Group 3: 7 (4 + 3)	NA	136 (26.2%)	NA	55 (26.6%)	NA	191 (26.3%)
Group 4: 8 (4 + 4, 3 + 5, 5 + 3)	NA	17 (3.3%)	NA	30 (14.5%)	NA	47 (6.5%)
Group 5: 9 or 10 (4 + 5, 5 + 4, or 5 + 5)	NA	25 (4.8%)	NA	29 (14.0%)	NA	54 (7.4%)
Mean PSA (ng/mL)	10.1	6.1	10.6	67.9	10.51	65.0

We successfully quantified mRNA expression of each biomarker in the 25-Gene Panel using preamplification of cDNA purified from urine pellets followed by real-time qRT-PCR. The retrospective cohort (*n* = 614) was used as a training set to create the Diagnosis Algorithm, which combined the mRNA expression quantity of the biomarkers in the panel for classification of the urine sample as PCa or benign prostate. Such diagnosis was then compared to the pathological diagnosis from biopsy to calculate the diagnostic performance of the 25-Gene Panel urine test.

**Fig. 2:**
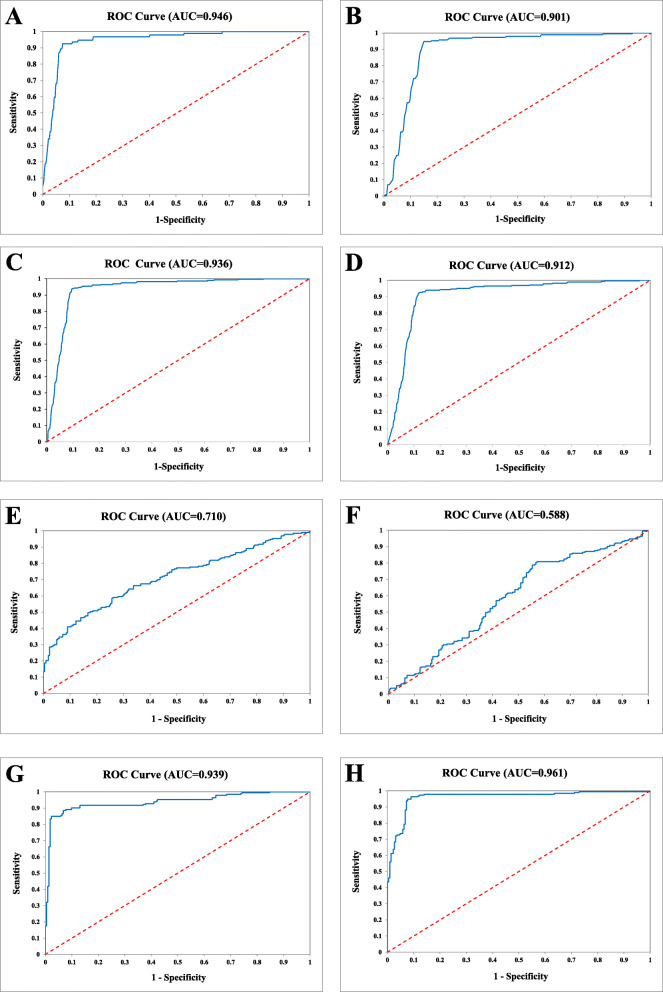
Receiver operating characteristic (ROC) curves for PCa diagnosis. ROC curve of the 25-Gene Panel urine test for PCa diagnosis in the retrospective training cohort (**a**), in the prospective validation cohort (**b**), and in the combination cohort (**c**); ROC curve of cross-validation of the 25-Gene Panel urine test for PCa diagnosis in the combination cohort (**d**); ROC curve of PSA (**e**), PSA at 4 ng/mL cutoff (**f**), the 25-Gene Panel urine test (**g**), and the 25-Gene Panel urine test and PSA combination (**h**) for PCa diagnosis in the cohort of 414 patients

As shown in Table 3 and Fig. 2a, the 25-Gene Panel was capable of distinguishing PCa from benign prostate (non-PCa) with high sensitivity of 92.5% (95% CI 94.8–90.2%), specificity of 91.5% (95% CI 97.1–85.9%), odds ratio of 132.6 (95% CI 293.5–59.9), and AUC of 0.946 (95% CI 0.963–0.929).

**Table 3:** Diagnostic performance of the 25-Gene Panel urine test in a retrospective training cohort (*n* = 614), a prospective validation cohort (*n* = 396), a combination cohort (*n* = 1010), and cross-validation of the combination cohort (*n* = 1010)

	Retrospective cohort			Prospective cohort			Combination cohort			Cross-validation		
	Positive	Negative	Total	Positive	Negative	Total	Positive	Negative	Total	Positive	Negative	Total
PCa	481	39	520	176	31	207	657	70	727	644	83	727
Non-PCa	8	86	94	10	179	189	18	265	283	27	256	283
Total	489	125	614	186	210	396	675	335	1010	671	339	1010
Sensitivity (95% CI)	92.5% (94.8–90.2%)			85.0% (89.9–80.2%)			90.4% (92.5–88.2%)			88.6% (90.9–86.3%)		
Specificity (95% CI)	91.5% (97.1–85.9%)			94.7% (97.9–91.5%)			93.6% (96.5–90.8%)			90.5% (93.9–87.0%)		
PPV (95% CI)	98.4% (99.5–97.2%)			94.6% (97.9–91.4%)			97.3% (98.6–96.1%)			96.0% (97.5–94.5%)		
NPV (95% CI)	68.8% (76.9–60.7%)			85.2% (90.0–80.4%)			79.1% (83.5–74.8%)			75.5% (80.1–70.9%)		
Odds ratio (95% CI)	132.6 (293.5–59.9)			101.6 (213.5–48.4)			138.2 (236.5–80.8)			73.6 (116.3–46.6)		

*PPV* positive predictive value, *NPV* negative predictive value, *CI* confidence interval

We then used an independent multi-center prospective cohort (*n* = 396) as a validation set to assess the diagnostic accuracy of the 25-Gene Panel urine test. The result showed sensitivity of 85.0% (95% CI 89.9–80.2%), specificity of 94.7% (95% CI 97.9–91.5%), odds ratio of 101.6 (95% CI 213.5–48.4), and AUC of 0.901 (95% CI 0.929–0.873) (Table 3 and Fig. 2b). The diagnostic performance was further validated by combining the retrospective (*n* = 614) and prospective (*n* = 396) cohorts, which used the same inclusion and exclusion criteria to enroll patients and collected urine samples without DRE, to form a combination cohort of 1010 patients with 283 benign prostate (28.0%) and 727 PCa (72.0%). The 25-Gene Panel showed high sensitivity of 90.4% (95% CI 92.5–88.2%), specificity of 93.6% (95% CI 96.5–90.8%), odds ratio of 138.2 (95% CI 236.5–80.8), and AUC of 0.936 (95% CI 0.956–0.916) (Table 3 and Fig. 2c). Cross-validation of the 25-Gene Panel urine test in the combination cohort generated similarly accurate diagnostic measures (Table 3 and Fig. 2d), further proving its accuracy in cancer diagnosis. These results from independent multi-center studies have clearly demonstrated the 25-Gene Panel urine test as an accurate tool to distinguish PCa and benign prostate. This suggests that the non-invasive and accurate urine test can be used to aid PCa diagnosis so only patients diagnosed to have PCa by the 25-Gene Panel urine test need to undergo biopsy to confirm the diagnosis.

### Comparison of the diagnostic performance of the 25-Gene Panel urine test with PSA and risk factors

Since PSA has been widely used as a PCa screening test, and age and PCa family history are risk factors for cancer, we compared the diagnostic performance of pre-biopsy PSA, PSA at 4 ng/mL cutoff value (commonly used cutoff for further testing in PCa screening) (PSA-4), age, and PCa family history (FH) with the 25-Gene Panel urine test (25-Gene) in patients from the combination cohort who had PSA test result or family history information. The patient cohort with PSA test result (referred as PSA Cohort) (*n* = 411) did not overlap with the patient cohort with family history information (referred as FH Cohort) (*n* = 451); thus, PSA and PSA-4 were assessed in the PSA Cohort while FH was assessed in the FH Cohort. Age and the 25-Gene Panel urine test were assessed in both PSA Cohort and FH Cohort. The 25-Gene Panel urine test had much higher accuracy in distinguishing PCa and benign prostate than PSA, PSA-4, age, and FH as shown by their respective *p* value, odds ratio, and AUC in univariate logistic regression analysis (*p* < 0.0001) (Table 4). PSA at 4 ng/mL cutoff is widely used in cancer screening, yet it had much lower specificity and AUC than the 25-Gene Panel urine test (30.2% vs 93.2% and 0.588 vs 0.939, respectively) (Table 5). This result demonstrated that the 25-Gene Panel urine test had superior diagnostic performance than PSA at 4 ng/mL, with greatly improved diagnostic specificity. Each year, more than 700,000 unnecessary negative biopsies were performed in the USA due to ~ 70% false positive rate of PSA at 4 ng/mL in the cancer screening test [24]. If the 25-Gene Panel urine test was used after the PSA test to determine the necessity of subsequent biopsy, the unnecessary biopsies could be reduced by 10-fold to avoid 630,000 biopsies in the USA alone, which could greatly reduce patient suffering and lower medical cost.

**Table 4:** Diagnostic performance of PSA, risk factors, the 25-Gene Panel urine test, and their combinations for PCa diagnosis in the PSA Cohort and FH Cohort

**PSA, age, and 25-Gene Panel in the PSA Cohort (*n*** **= 414)**												
	**Univariate**			**Multivariate (age, PSA, 25-Gene)**			**Multivariate (PSA, 25-Gene)**			**Multivariate (age, 25-Gene)**		
	*p* value	OR (95% CI)	AUC (95% CI)	*p* value	OR (95% CI)	AUC (95% CI)	*p* value	OR (95% CI)	AUC (95% CI)	*p* value	OR (95% CI)	AUC (95% CI)
Age	0.46	0.5 (2.4–0.1)	0.516 (0.572–0.460)	0.20	1.0 (1.1–1.0)	–	–	–	–	0.08	1.0 (1.0–1.1)	–
PSA	< 0.0001	6.9 (12.2–3.9)	0.710 (0.759–0.661)	< 0.0001	1.1 (1.1–1.0)	–	< 0.0001	1.1 (1.0–1.1)	–	–	–	–
25-Gene	< 0.0001	107.3 (213.2–54.0)	0.939 (0.962–0.916)	< 0.0001	281.8 (741.9–107.0)	–	< 0.0001	255.0 (644.1–101.0)	–	< 0.0001	116.2 (237.1–57.0)	–
Combo	–	–	–	< 0.0001	194.5 (429.1–88.1)	0.967 (0.984–0.950)	< 0.0001	195.5 (431.4–88.6)	0.961 (0.980–0.942)	< 0.0001	106.6 (212.0–53.7)	0.923 (0.949–0.897)
**PSA at 4 ng/mL cutoff, age, and 25-Gene Panel in the PSA Cohort (*n*** **= 414)**												
	**Univariate**			**Multivariate (age, PSA-4, 25-Gene)**			**Multivariate (PSA-4, 25-Gene)**					
	*p* value	OR (95% CI)	AUC (95% CI)	*p* value	OR (95% CI)	AUC (95% CI)	*p* value	OR (95% CI)	AUC (95% CI)			
Age	0.46	0.5 (2.4–0.1)	0.516 (0.572–0.460)	0.08	1.0 (1.1–1.0)	–	–	–	**–**			
PSA-4	0.001	2.3 (3.7–1.4)	0.588 (0.642–0.534)	0.20	1.7 (3.8–0.8)	–	0.21	1.7 (3.8–0.7)	**–**			
25-Gene	< 0.0001	107.3 (213.2–54.0)	0.939 (0.962–0.916)	< 0.0001	112.6 (230.0–55.1)	–	< 0.0001	103.9 (206.7–52.2)	**–**			
Combo	–	–	–	< 0.0001	106.6 (212.0–53.7)	0.927 (0.953–0.901)	< 0.0001	107.3 (213.2–54.0)	0.942 (0.965–0.919)			
**PCa family history, age, and 25-Gene Panel in the FH Cohort (*n*** **= 451)**												
	**Univariate**			**Multivariate (age, PCa FH, 25-Gene)**			**Multivariate (PCa FH, 25-Gene)**			**Multivariate (age, 25-Gene)**		
	*p* value	OR (95% CI)	AUC (95% CI)	*p* value	OR (95% CI)	AUC (95% CI)	*p* value	OR (95% CI)	AUC (95% CI)	*p* value	OR (95% CI)	AUC (95% CI)
Age	0.02	0.9 (1.0–0.9)	0.606 (0.706–0.506)	0.12	0.9 (1.0–0.7)	–	–	–	**–**	0.13	0.9 (1.0–0.8)	–
PCa FH	0.28	2.0 (6.6–0.6)	0.363 (0.448–0.278)	0.23	5.0 (67.1–0.4)	–	0.27	3.9 (45.6–0.3)	**–**	–	–	–
25-Gene	< 0.0001	3690.0 (33,894.8–401.7)	0.985 (1.013–0.957)	< 0.0001	5760.4 (74,490.3–445.5)	–	< 0.0001	4080.0 (39,436.1–422.1)	**–**	< 0.0001	4530.3 (48,645.8–421.9)	–
Combo	–	–	–	< 0.0001	3690.0 (33,894.8–401.7)	0.987 (1.013–0.961)	< 0.0001	3690.0 (33,894.8–401.7)	0.987 (1.013–0.961)	< 0.0001	3690.0 (33,894.8–401.7)	0.986 (1.013–0.959)

*25-Gene* 25-Gene Panel, *OR* odds ratio, *AUC* area under the ROC curve, *CI* confidence interval, *PSA-4* PSA at 4 ng/mL cutoff, *FH* family history, *Combo* combination

**Table 5:** Comparison of diagnostic performance of PSA, PSA at 4 ng/mL cutoff, age, and the 25-Gene Panel urine test and their combinations for PCa diagnosis in the PSA Cohort

	Sensitivity (95% CI)	Specificity (95% CI)	PPV (95% CI)	NPV (95% CI)	OR (95% CI)	AUC (95% CI)
PSA	36.3% (43.1–29.5%)	92.3% (94.8–88.8%)	80.5% (88.8–72.1%)	62.5% (67.7–57.3%)	6.7 (12.2–3.9)	0.710 (0.759–0.661)
PSA-4	83.9% (89.1–78.8%)	30.2% (36.2–24.1%)	51.1% (56.6–45.6%)	68.4% (77.6–59.2%)	2.3 (3.7–1.4)	0.588 (0.642–0.534)
Age	1.0% (2.5–0.4%)	97.8% (99.7–95.8%)	28.6% (62.0–4.9%)	53.3% (58.2–48.5%)	0.5 (2.4–0.1)	0.516 (0.572–0.460)
25-Gene	88.6% (93.1–84.1%)	93.2% (96.6–90.0%)	91.9% (95.9–88.0%)	90.4% (94.2–86.6%)	107.3 (213.2–54.0)	0.939 (0.962–0.916)
PSA+25-Gene	94.8% (98.0–91.7%)	91.4% (95.1–87.8%)	90.6% (94.6–86.6%)	95.3% (98.2–92.5%)	195.5 (431.4–88.6)	0.961 (0.980–0.942)
PSA-4+25-Gene	88.6% (93.1–84.1%)	93.2% (96.6–90.0%)	91.9% (95.9–88.0%)	90.4% (94.2–86.6%)	107.3 (213.2–54.0)	0.942 (0.965–0.919)
PSA+Age+25-Gene	94.8% (97.9–91.7%)	91.4% (95.1–87.8%)	90.6% (94.6-86.5%)	95.3% (98.2–94.5%)	194.5 (429.1–88.1)	0.967 (0.984–0.950)
PSA-4+Age+25-Gene	88.5% (93.1–84.0%)	93.2% (96.6–89.9%)	91.9% (95.8–88.0%)	90.4% (94.2–86.6%)	106.6 (212.0–53.7)	0.927 (0.953–0.901)

*PPV* positive predictive value, *NPV* negative predictive value, *OR* odds ratio, *AUC* area under the ROC curve, *CI* confidence interval, *PSA-4* PSA at 4 ng/mL cutoff

In addition, to examine if PSA and the risk factors could be combined with the 25-Gene Panel urine test to enhance its diagnostic performance, various combinations were assessed by multivariate logistic regression analysis. The result showed that when the 25-Gene Panel urine test was combined with PSA (25-Gene+PSA) in the PSA Cohort, both the odds ratio and AUC were significantly increased (odds ratio of 107.3 (95% CI 213.2–54.0) and AUC of 0.939 (95% CI 0.962–0.916) for the 25-Gene Panel alone vs odds ratio of 195.5 (95% CI 431.4–88.6) and AUC of 0.961 (95% CI 0.980–0.942) for 25-Gene+PSA) (*p* < 0.01) (Table 4, Fig. 2h). However, combination of the 25-Gene Panel urine test with age (25-Gene+age) in the PSA Cohort, combination of the 25-Gene Panel urine test with family history (25-Gene+FH) in the FH Cohort, or combination of the 25-Gene Panel urine test with PSA-4 (25-Gene+PSA-4) in the PSA Cohort did not significantly alter the diagnostic accuracy of the 25-Gene Panel urine test, as neither odds ratio nor AUC differ significantly in these combinations (Table 4). Furthermore, important diagnostic measures including sensitivity, specificity, positive predictive value (PPV), and negative predictive value (NPV) of the 25-Gene Panel urine test combined with PSA, PSA plus age, PSA-4, and PSA-4 plus age in the PSA Cohort were compared. As shown in Table 5, 25-Gene+PSA had higher accuracy than 25-Gene alone, 25-Gene+PSA-4, or 25-Gene+PSA-4+age, with exceptionally high sensitivity of 94.8% (95% CI 98.0–91.7%), specificity of 91.4% (95% CI 95.1–87.8%), PPV of 90.6% (95% CI 94.6–86.6%), and NPV of 95.3% (95% CI 98.2–92.5%). The addition of age to 25-Gene+PSA did not change its diagnostic accuracy except for a slight increase of AUC (0.961 (95% CI 0.980–0.942) vs 0.967 (95% CI 0.984–0.950)) (*p* > 0.05). These results suggest that the 25-Gene Panel urine test can be combined with pre-biopsy PSA to provide more accurate cancer diagnosis.

### In silico validation of the 25-Gene Panel for cancer diagnosis

To validate the differential gene expression of the 25 genes in the panel in PCa and benign prostate tissue specimens, we used a prostate tissue cohort GSE17951 (*n* = 154) obtained from the GEO database (NCBI) (17, 18). The mRNA expression data of the 25 genes was obtained from the database and normalized with beta-actin expression. A large group of the genes including *HIF1A* (*p* = 0.013), *BIRC5* (*p* < 0.0001), *AMACR* (*p* < 0.0001), *CRISP3* (*p* < 0.0001), *HPN* (*p* < 0.0001), *MYO6* (*p* < 0.0001), *GOLM1* (*p* < 0.0001), *LMTK2* (*p* < 0.001), *EZH2* (*p* < 0.0001), *PCA3* (*p* < 0.0001), *PIP5K1A* (*p* < 0.0001), *CDK1* (*p* < 0.0001), *ANXA3* (*p* = 0.008), *CCND1* (*p* = 0.012), and *KLK3* (*p* < 0.0001) had significantly increased expression in PCa specimens as compared with that of benign prostate, while a small group of genes including *FGFR1* (*p* = 0.286), *TMPRSS2* (*p* = 0.369), *VEGFA* (*p* = 0.464), and *FN1* (*p* = 0.632) had statistically insignificant increase in gene expression (Additional file 3: Fig. S1). In contrast, several genes including *PMP22* (*p* < 0.0001), *GSTP1* (*p* < 0.0001), and *CST3* (*p* < 0.0001) had significantly decreased expression in PCa specimens as compared with that of benign prostate, and a few genes including *CCNA1* (*p* = 0.112), *PSCA* (*p* = 0.187), and *PTEN* (*p* = 0.493) had statistically insignificant decrease in gene expression (Additional file 3: Fig. S2). When the 25 genes were combined as a panel, the discriminant score F1 showed strikingly higher level in PCa than that in benign prostate (*p* < 0.0001) (Additional file 3: Fig. S3). In addition, the diagnostic performance of the 25-Gene Panel to distinguish PCa and benign prostate was assessed in the GSE17951 cohort and the result showed very high sensitivity of 100% (95% CI 100–100%), specificity of 96.0% (95% CI 99.8–92.0%) (Additional file 2: Table S4), and AUC of 0.998 (95% CI 1.004–0.992) (Additional file 3: Fig. S4). The in silico study result confirmed the results from the urine study for the high diagnostic accuracy of the 25-Gene Panel.

### Identification of clinically significant cancer

**Fig. 3:**
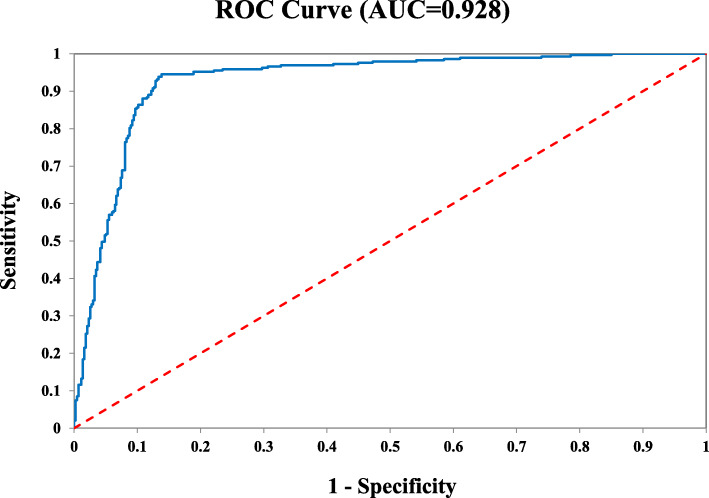
Receiver operating characteristic (ROC) curve of the 25-Gene Panel urine test for the identification of clinically significant PCa in cancer patients from the retrospective and prospective cohorts (*n* = 727)

It is important to develop accurate tests to identify and subtype clinically significant and insignificant PCa. We examined whether the 25-Gene Panel urine test could be used to identify clinically significant PCa. In the retrospective and prospective cohorts, 727 patients were diagnosed to have PCa by routine biopsy. Using the 25-Gene Panel urine test with a Stratification Algorithm (Additional file 1: Methods), clinically significant and insignificant PCa were identified with high accuracy as shown by AUC of 0.928 (95% CI 0.947–0.909) (Fig. 3). Such an accurate and convenient urine test may be used to identify clinically significant cancer patients for immediate treatment. For patients with clinically insignificant cancer, it can be used periodically to monitor cancer progression during active surveillance.

### Preliminary study to test the 25-Gene Panel urine test for prostatectomy treatment follow-up

Currently, no accurate method is available to check if radical prostatectomy (RP) has completely removed prostate tumors. To test if the 25-Gene Panel urine test could be used to show the absence of PCa after the tumors had been surgically removed by RP, we collected urine from ten patients before and after RP and performed diagnosis using the 25-Gene Panel. As shown in Table 6, nine out of ten urine samples (90%) were diagnosed to be non-PCa after RP, which was consistent with successful RP in most patients. The one patient diagnosed to be PCa may still have residual cancer lesion after the surgery and need additional treatment. The result of the preliminary study in the small patient cohort suggests that the 25-Gene Panel urine test has potential to be used as an accurate and simple method to measure efficacy of RP for treatment follow-up.

**Table 6:** Diagnosis of pre- and post-prostatectomy urine samples by the 25-Gene Panel urine test

	Pre-surgery urine	Post-surgery urine
Patient A	PCa	PCa
Patient B	PCa	Non-PCa
Patient C	PCa	Non-PCa
Patient D	PCa	Non-PCa
Patient E	PCa	Non-PCa
Patient F	PCa	Non-PCa
Patient G	PCa	Non-PCa
Patient H	PCa	Non-PCa
Patient I	PCa	Non-PCa
Patient J	PCa	Non-PCa
% Non-PCa	0	90.0%

### The 25-Gene Panel urine test is PCa-specific

In the urine cohorts, some patients had other types of cancers in addition to PCa or benign prostate (Table 2), especially urinary tract cancers such as bladder cancer, which might affect PCa diagnosis since cells of other cancers could be released into the urine. We have not found any study addressing this issue; therefore, we tested if the presence of other cancers could affect diagnosis of the 25-Gene Panel urine test. We found that all of the PCa patients who also had other types of cancers (two had bladder cancer, one each had melanoma, kidney and colorectal cancer) were diagnosed to have PCa, while all of the benign prostate patients with other cancers (one had bladder cancer, one each had lung and skin cancer) were diagnosed to be non-PCa. This suggests that our test was specific for PCa diagnosis without being affected by the presence of other cancers.

## Discussion

In this study, we have developed a novel 25-Gene Panel urine test that can be used for PCa diagnosis to accurately identify patients who need to have biopsy to avoid large amount of unnecessary biopsies each year. In addition, it can be used as an accurate and non-invasive test to identify clinically significant and insignificant cancer to assist treatment decision and active cancer surveillance. Further, it may potentially be used as a treatment follow-up test to assess if residual cancer exists after prostatectomy or other cancer therapies to determine if further treatment is necessary. The 25-Gene Panel urine test was found to be specific for PCa diagnosis, even for patients with other types of cancers. Lastly, the non-invasive and convenient urine test without DRE may be performed by patients at home to facilitate cancer surveillance and post-treatment follow-up.

The study population in the retrospective and prospective cohorts represented patients in real clinical practice as they were from the clinical cases obtained from the participating hospitals. These patients with elevated PSA underwent scheduled biopsy for cancer diagnosis/treatment. AUC analysis is an important tool to assess the diagnostic performance of the 25-Gene Panel. In addition, other important parameters including sensitivity, specificity, positive predictive value, negative predictive value, and odds ratio were used to assess the 25-Gene Panel. Thus, combining these measurements provided valid assessment of the 25-Gene Panel urine test.

Currently, none of the clinical parameters (i.e., PSA and its derivatives such as PHI), biomarkers (i.e., PCA3), or combinations of biomarkers or clinical parameters (i.e., PCA3 combined with TMPRSS2:ERG, microRNA signatures, metabolomic biomarkers) used in clinical practice or reported in publications was able to diagnose PCa or stratify cancer risk with > 90% sensitivity and specificity, and AUC over 0.9, as shown in several recent reviews [2, 4–6, 8–10, 25–27]. Our 25-Gene Panel urine test was validated for accurate cancer diagnosis by two independent multi-center study cohorts as well as the large combination cohort with uniformly high diagnostic sensitivity and specificity above 90% and AUC exceeding 0.9. In statistics, AUC of the ROC curve is an important measure of how accurate a classifier can predict future classification, and AUC over 0.9 indicates an accurate classifier [28]. The fact that the AUC values of the 25-Gene Panel urine test in all cohorts were well above 0.9 suggests it may be a more accurate and superior PCa diagnostic tool than PSA, clinical parameters, existing biomarkers, and their combinations. Our study found that the 25-Gene Panel urine test could be combined with PSA to provide exceptionally accurate diagnosis. In clinical practice, it may be combined with PSA, multiparametric MRI imaging, and biopsy to greatly improve diagnostic accuracy and avoid unnecessary biopsy and overdiagnosis.

For cancer diagnosis and treatment, it is important to identify clinically significant and insignificant cancer so patients with clinically significant cancer are given immediate treatment while clinically insignificant cancer patients are placed under active surveillance. In our study, we found that the 25-Gene Panel was able to accurately identify clinically significant and insignificant cancers. Thus, the 25-Gene Panel has great potential to improve cancer diagnosis and treatment.

In this study, the diagnostic performance of the 25-Gene Panel in the retrospective and prospective cohorts were similar, regardless of using freshly collected urine or frozen urine pellet stored for long term. In addition, the PCa patients in the retrospective cohort had a mean PSA level of 6.1 ng/mL, while the patients in the prospective cohort had a high average PSA level of 67.9 ng/mL (Table 1). This showed that the diagnostic performance of the 25-Gene Panel was not affected by high PSA levels.

The similar diagnostic performance obtained in the cohorts consisting of patients with different ethnic background (Caucasians in the retrospective cohort and Asians in the prospective cohort) and clinical characteristics (such as different PSA levels and Gleason scores) suggests that the test is robust and may be used in different patient populations regardless of race, ethnic background, or clinical characteristics.

A small number of urine samples were excluded from the study due to little or no prostate cells collected in the urine. We tested and found that the first morning urine with at least 45 mL volume, especially the early stream, contained sufficient amount of urine cells for mRNA quantification (data not shown), thus can be used to solve this problem. Since no DRE is necessary and the urine can be stored at room temperature for a week with the DNA/RNA preservative, collecting first morning urine sample is practical for clinical practice. Our non-invasive urine test without DRE that can use urine collected by patients at home represents a novel and significantly improved method for PCa diagnosis and prognosis.

The 25-Gene Panel consists of several known PCa-specific biomarkers (*PCA3*, *TMPRSS2*); biomarkers with potential diagnostic or prognostic values (*ANXA3*, *CRISP3*, *CST3*, *KLK3*, *PSCA*, *EZH2*, *GSTP1*, *AMACR*); biomarkers associated with cellular functions including proliferation, survival, migration, and metastasis (*FGFR1*, *CCNA1*, *CDK1*, *CCND1*, *HIF1A*, *HPN*, *VEGFA*, *PTEN*, *PIP5K1A*); and biomarkers whose involvement in cancer remains unknown (*LMTK2*, *MYO6*, *BIRC5*, *FN1*, *GOLPH2*, *PMP22*) [29–34].

One of the limitations of this study was that there were much less benign prostate urine samples (15.31%) than PCa urine samples (84.69%) in the retrospective cohort, and as a consequence, less benign prostate (28.00%) than PCa (72.00%) samples in the combined cohort. This was due to that less archived benign prostate patient samples were available for our study. The imbalance of the two classes may not reflect the real clinical situation and could theoretically affect the diagnostic measures, resulting in higher sensitivity and PPV, and lower specificity and NPV. However, since the prospective cohort with more balanced benign prostate and PCa samples (47.73% for benign prostate and 52.27% for PCa) had similar diagnostic performance as the retrospective cohort except for higher NPV, it suggests that the effect of the imbalance was limited. Moreover, the AUC of these cohorts were all above 0.9, which suggests that the urine test had similarly high diagnostic accuracy in all cohorts. Nevertheless, it would be better to have a cohort with the number of benign prostate and PCa patients reflecting patient composition in real clinical settings. Thus, more prospective studies will be conducted in the future to further validate the 25-Gene Panel urine test. Another limitation is only a small portion of patients in the retrospective cohort had PSA test result and little cancer staging information was available in the prospective cohort. Thus, large prospective studies with collection of more patient information will be conducted in the future to further validate the 25-Gene Panel urine test and assess its combination with other PCa diagnostic methods such as PSA and MRI imaging. Further, the preliminary study to assess the ability of the 25-Gene Panel urine test to detect if RP has removed cancer lesion was conducted in a small subset of patients who underwent RP, thus future studies with large patient cohorts are needed and will be conducted to determine if the 25-Gene Panel urine test can be used for cancer treatment monitoring.

## Conclusions

In summary, we have developed and validated a highly accurate and non-invasive 25-Gene Panel urine test as the next-generation liquid biopsy method for PCa diagnosis and potential treatment follow-up to improve cancer diagnosis and treatment.

## Supplementary Information

**Additional file 1.** Supplementary methods, including quantification of mRNA expression, validation of urine test without DRE, Algorithm for identification of clinically significant cancer, and statistical analysis.

**Additional file 2:** Supplementary tables, including comparison of Ct values of three genes with and without preamplification before real time qRT-PCR (**Table S1**), comparison of CtS values of five genes in the urine samples collected with and without digital rectal examination (DRE) (**Table S2**), genes in the 25-Gene Panel for prostate cancer diagnosis (**Table S3**), diagnostic performance of the 25-Gene Panel in GSE17951 prostate tissue specimen cohort (*n* = 154) (**Table S4)**.

**Additional file 3.** Supplementary figures, including box plots of biomarkers with increased gene expression levels in prostate tissue specimens from patients with prostate cancer as compared to patients with benign prostate in the GSE17951 cohort (*n* = 154) (**Fig. S1**), box plots of biomarkers with decreased gene expression levels in prostate tissue specimens from patients with prostate cancer as compared to patients with benign prostate in the GSE17951 cohort (*n* = 154) (**Fig. S2**), box plot of discriminant score F1 of the 25-Gene Panel in prostate tissue specimens from patients with prostate cancer as compared to patients with benign prostate in the GSE17951 cohort (n = 154) (**Fig. S3**), and receiver operating characteristic (ROC) curve of the 25-Gene Panel for PCa diagnosis in GSE17951 prostate tissue specimen cohort (*n* = 154) **(Fig. S4**).

## Data Availability

The data obtained and analyzed in this study are included in the manuscript.

## Abbreviations

AUC: Area under the curve
CHTN: Cooperative Human Tissue Network
CI: Confidence interval
Ct: Cycle threshold
CtS: Relative cycle threshold
DA: Discriminant analysis
DRE: Digital rectal examination
FH: Family history
GEO: Gene Expression Omnibus
IRB: Institutional Review Board
NPV: Negative predictive value
OR: Odds ratio
PCa: Prostate cancer
PPV: Positive predictive value
PSA: Prostate-specific antigen
ROC: Receiver operating curve
RP: Radical prostatectomy
