# Supplementary material for: Development and validation of a 25-Gene Panel urine test for prostate cancer diagnosis and potential treatment follow-up
Source: BMC Med. 2020 Dec 1;18:376. doi: 10.1186/s12916-020-01834-0 (PMC7706045; doi:10.1186/s12916-020-01834-0)
Supplement: Supplementary file 1 — Additional file 1. Supplementary methods, including quantification of mRNA expression, validation of urine test without DRE, Algorithm for identification of clinically significant cancer, and statistical analysis. [file 12916_2020_1834_MOESM1_ESM.docx]

**Development and Validation of a 25-Gene Panel Urine Test for Prostate Cancer Diagnosis and Potential Treatment Follow-up**

**Additional File 1**

**Supplementary Methods**

**Quantification of mRNA expression**

The frozen urine pellet was thawed at 37°C and resuspended in cold PBS followed by centrifugation at 1000 ×g for 10 min. Quick-RNA MicroPrep Kit was used to purify total RNA from the cell pellet following the manufacturer’s procedure (Zymo Research, Irvine, CA, USA). 100 ng purified RNA was then used for reverse transcription of cDNA using either High Capacity cDNA Reverse Transcription Kit (Life Technologies, Foster City, CA, USA) or iScript Reverse Transcription Supermix for real time qRT-PCR (Bio-Rad, Hercules, CA, USA) following the manufacturers’ protocols. The cDNA from reverse transcription was preamplified using TaqMan® PreAmp Master Mix (Thermo Fisher Scientific, Waltham, MA, USA) or PCa PreAmplification Mix (Hao Rui Jia Biotech Ltd., Beijing, China) according to the manufacturers’ directions. Real-time qRT-PCR was performed to assess mRNA expression levels using predesigned primers and probe assays from Integrated DNA Technologies (San Diego, CA, USA). Real-time qRT-PCR was performed on ABI Quantstudio 6, ABI 7500 or ABI 7900 Real-Time PCR System (Thermo Fisher Scientific, Waltham, MA, USA). The PCR reaction was set in 10 μl volume, which contains preamplified cDNA transcribed from 0∙2 ng of purified RNA, 5 μl of 2x TaqMan® Universal PCR Master Mix (Thermo Fisher Scientific, Waltham, MA, USA) or PrimeTime® Gene Expression Master Mix (Integrated DNA Technologies, San Diego, CA, USA), 500 nM each of forward and reverse amplification primers, and 250 nM of probe. The real-time qRT-PCR was performed using the following cycling condition: 10 minutes at 95°C for polymerase activation, and 40 cycles of 15 seconds at 95°C and 1 minute at 60°C. For each gene, triplicate PCR were performed.

**Validation of urine test without DRE**

A method of cDNA preamplification before real time qRT-PCR was used to detect gene expression at low levels in the urine without DRE containing fewer prostate cells. Expression values of three genes randomly chosen from the 25-Gene Panel in two patient urine samples were measured to test if the preamplification method could increase the ability of qRT-PCR to detect gene expression by without changing the relative gene expression values (ΔCt). In addition, Ct values of five genes in five patient urine samples on two days with and without digital rectal examination (DRE), respectively were assessed and the normalized Ct value of each gene in the urine with DRE (CtS DRE+) was compared to that in the urine without DRE (CtS DRE-) for each patient. Furthermore, the Diagnostic D Scores and diagnosis results for each patient using urine with or without DRE were obtained and compared.

**Algorithm for identification of clinically significant cancer**

To generate an algorithm for diagnosing clinically significant or insignificant PCa (Stratification Algorithm), discriminant analysis was performed to test the association between pathological diagnosis of clinically significant or insignificant PCa and CtS values of the 25 genes in the panel using a statistical software program XLSTAT (Addinsoft, Paris, France). The diagnosis of all the samples by the algorithm was compared to their pathological diagnosis to assess diagnostic performance such as AUC.

For diagnosis of clinically significant or insignificant PCa using urine samples, the CtS values of the 25 genes in the panel were used to generate a classification score (Stratification D Score) for each urine sample using the following algorithm:

C_HighRisk_=A_H_+CtS_1_*H_1_+CtS_2_*H_2…_+CtS_25_*H_25_+CtS_1_*CtS_1_*H_1*1_+CtS_1_*CtS_2_*H_1*2…_+CtS_1_*CtS_25_*H_1*25_+CtS_2_*CtS_2_*H_2*2…_+CtS_2_*CtS_25_*H_2*25…_+CtS_25_* CtS_25_*H_25*25_

C_LowRisk_=B_L_+CtS_1_*L_1_+CtS_2_*L_2…_+CtS_25_*L_25_+CtS_1_*CtS_1_*L_1*1_+CtS_1_*CtS_2_*L_1*2…_+CtS_1_*CtS_25_*L_1*25_+CtS_2_*CtS_2_*L_2*2…_+CtS_2_*CtS_25_*L_2*25…_+CtS_25_* CtS_25_*L_25*25_

Stratification D Score=C_HighRisk_-C_LowRisk_

Whereas A_H_ is clinically significant PCa constant, B_L_ is clinically insignificant PCa constant, CtS_1_ through CtS_25_ are CtS values of gene 1 through gene 25, H_1_ through H_25_ are clinically significant PCa regression coefficients of gene 1 through gene 25, H_1*1_ through H_25*25_ are gene 1 and gene 1 cross clinically significant PCa regression coefficients through gene 25 and gene 25 cross clinically significant PCa regression coefficients, L_1_ through L_25_ are clinically insignificant PCa regression coefficients of gene 1 through gene 25, and L_1*1_ through L_25*25_ are gene 1 and gene 1 cross clinically insignificant PCa regression coefficients through gene 25 and gene 25 cross clinically insignificant PCa regression coefficients. Each gene in the panel is important for the algorithm. The sample was diagnosed to be clinically significant PCa when stratification D Score was >0, whereas the sample was diagnosed to be clinically insignificant PCa when stratification D Score was ≤0.

**Statistical analysis**

For CtS values of five genes in each patient, the p values were calculated using two-tailed test in Excel program. In the gene expression validation study, box plot analysis was performed in a statistical software program XLSTAT (Addinsoft, Paris, France) by using the normalized mRNA expression data of each gene in the 25-Gene Panel in PCa and Non-PCa prostate tissue specimens from the GSE17951 cohort. The nonparametric Mann–Whitney test was used to assess statistical significance of the differential gene expression in PCa and Non-PCa specimens..
