# Supplementary material for: Development and validation of a 25-Gene Panel urine test for prostate cancer diagnosis and potential treatment follow-up
Source: BMC Med. 2020 Dec 1;18:376. doi: 10.1186/s12916-020-01834-0 (PMC7706045; doi:10.1186/s12916-020-01834-0)
Supplement: Supplementary file 2 — Additional file 2: Supplementary tables, including comparison of Ct values of three genes with and without preamplification before real time qRT-PCR (Table S1), comparison of CtS values of five genes in the urine samples collected with and without digital rectal examination (DRE) (Table S2), genes in the 25-Gene Panel for prostate cancer diagnosis (Table S3), diagnostic performance of the 25-Gene Panel in GSE17951 prostate tissue specimen cohort (n = 154) (Table S4). [file 12916_2020_1834_MOESM2_ESM.docx]

**Development and Validation of a 25-Gene Panel Urine Test for Prostate Cancer Diagnosis and Potential Treatment Follow-up**

**Additional File 2**

**Supplementary Tables**

**Table S1** Comparison of Ct values of three genes with and without preamplification before real time qRT-PCR

|  | **Sample 807** | | | | | **Sample 301** | | | |
| --- | --- | --- | --- | --- | --- | --- | --- | --- | --- |
|  | **No Amp** | **Preamp** | | **Difference** | **SD** | **No Amp** | **Preamp** | **Difference** | **SD** |
| CtGene A | 26.0 | 16.5 | | 9.5 | 0.01 | 26.1 | 16.0 | 10.2 | 0.5 |
| CtGene B | 35.1 | 25.3 | | 9.9 |  | 33.9 | 24.5 | 9.5 |  |
| CtGene C | 35.3 | 25.8 | | 9.5 |  | 29.8 | 20.3 | 9.4 |  |
| ΔCtGene B | 9.1 | 8.8 | 0.3 | | 0.3 | 7.8 | 8.5 | -0.7 | 0.01 |
| ΔCtGene C | 9.2 | 9.3 | | -0.02 |  | 3.7 | 4.4 | -0.7 |  |

ΔCtGene B: CtGene B-CtGene A; ΔCtGene C: CtGene C-CtGene A; No Amp: no preamplification of cDNA before qRT-PCR; Preamp: preamplification of cDNA before qRT-PCR; SD: standard deviation.

**Table S2** Comparison of CtS values of five genes in the urine samples collected with and without digital rectal examination (DRE)

|  |  | **Gene A** | **Gene B** | **Gene C** | **Gene D** | **Gene E** | **P Value** |
| --- | --- | --- | --- | --- | --- | --- | --- |
| Patient 1 | CtS DRE- | 1274.2 | 1271.2 | 1340.1 | 1310.8 | 1061.5 | 0.8 |
|  | CtS DRE+ | 1258.0 | 1242.5 | 1343.1 | 1286.2 | 1057.7 |  |
|  | SD (SD/Mean) | 11.4 (0.9%) | 20.31 (1.6%) | 2.1 (0.16%) | 17.42 (1.3%) | 2.72 (0.3%) |  |
| Patient 2 | CtS DRE- | 1278.2 | 1224.7 | 1559.2 | 1268.2 | 1063.8 | 0.5 |
|  | CtS DRE+ | 1216.3 | 1200.2 | 1368.0 | 1243.5 | 1056.6 |  |
|  | SD (SD/Mean) | 43.8 (3.5%) | 17.3 (1.4%) | 135.2 (9.2%) | 17.5 (1.4%) | 5.1 (0.5%) |  |
| Patient 3 | CtS DRE- | 1230.2 | 1154.6 | 1258.8 | 1187.3 | 1043.5 | 0.8 |
|  | CtS DRE+ | 1178.4 | 1163.7 | 1239.6 | 1191.4 | 1047.4 |  |
|  | SD (SD/Mean) | 36.7 (3.1%) | 6.5 (0.6%) | 13.6 (1.1%) | 2.9 (0.2%) | 2.8 (0.3%) |  |
| Patient 4 | CtS DRE- | 1475.8 | 1390.8 | 1814.4 | 1805.3 | 1808.7 | 0.5 |
|  | CtS DRE+ | 1448.0 | 1406.9 | 1792.4 | 1608.6 | 1589.4 |  |
|  | SD (SD/Mean) | 19.7 (1.4%) | 11.4 (0.8%) | 15.6 (0.9%) | 139.1 (8.2%) | 155.1 (9.1%) |  |
| Patient 5 | CtS DRE- | 1189.3 | 1255.0 | 1471.8 | 1287.7 | 1051.5 | 0.7 |
|  | CtS DRE+ | 1223.9 | 1206.2 | 1346.6 | 1247.4 | 1061.5 |  |
|  | SD (SD/Mean) | 24.5 (2.0%) | 34.46 (2.8%) | 85.55 (6.3%) | 28.5 (2.3%) | 7.1 (0.7%) |  |

CtS: Ct (sample)/Ct (actin)*1000; CtS DRE-: CtS of gene detected in the urine sample collected without DRE; CtS DRE+: CtS of gene detected in the urine sample collected after DRE; SD: standard deviation.

**Table S3** Genes in the 25-Gene Panel for prostate cancer diagnosis

*AMACR*: encodes alpha-methylacyl-CoA racemase, a mitochondrial and peroxisomal enzyme important for beta oxidation of branched-chain fatty acids and bile acid intermediates; expressed in prostate, colorectal, liver, melanomas, thyroid, gastric and renal cancers.

*ANXA3*: encodes annexin A3, a calcium-dependent phospholipid-binding protein that regulates cell growth and signal transduction pathways, and is involved in cancers such as colorectal, breast, lung and prostate.

*BIRC5*: encodes baculoviral IAP repeat containing 5, a negative regulatory protein that blocks apoptosis; expressed at high levels during fetal development and in most cancers.

*CCNA1*: encodes cyclin D1 that regulates CDK2 and CDC2 kinases at S and G2 phase of the cell cycle, and binds cell cycle regulators Rb, E2F-1, and p21; expressed in testis, brain, and several cancers such as endometrial, ovarian and prostate cancers.

*CCND1*: encodes cyclin D1, a mitotic cyclin that regulates CDK kinases and is required for cell cycle progression. Gene mutations and amplification are frequently found in many tumors.

*CDK1*: encodes a Ser/Thr kinase, part of a M-phase promoting factor essential for G1/S and G2/M phase transitions of the cell cycle, and its phosphorylation and dephosphorylation is important for regulating cell cycle; expressed in many types of cancers.

*CRISP3*: encodes cysteine rich secretory protein 3, a member of the CRISP protein family; expressed in the male reproductive tract and involved in sperm function and fertilization, and in the female reproductive tract involved in endometrial receptivity for embryo implantation; differentially expressed in aggressive and indolent PCa.

*CST3*: encodes cystatin C, an abundant extracellular inhibitor of cysteine proteases with a role in vascular disease; expressed in most cancers, and differentially expressed in aggressive and indolent PCa.

*EZH2*: encodes enhancer of zeste 2 polycomb repressive complex 2 subunit, a catalytic [subunit](https://en.wikipedia.org/wiki/Protein_subunit) of polycomb repressive complex 2, which is involved in transcriptional regulation of histone methylation; expression level correlated with PCa development and upregulated in metastatic PCa.

# *FGFR1*: encodes fibroblast growth factor receptor 1, which binds both acidic and basic fibroblast growth factors and is involved in limb induction; mutations associated with several diseases such as Pfeiffer syndrome; expressed in many cancers and defines different subtypes of pancreatic cancer; found to be important for prostate cancer progression and metastasis.

*FN1*: encodes fibronectin 1, an extracellular matrix protein involved in cell adhesion, migration, and cancer metastasis; expressed in all cancers.

*GOLPH2 (GOLM1)*: encodes Golgi membrane protein 1, which is important for sorting and modification of exported proteins from the endoplasmic reticulum; expression upregulated in many cancers including breast, cervical and prostate cancers; found to be involved in cancer progression and metastasis.

*GSTP1*: encodes glutathione S-transferase P1, a member of Glutathione S-transferases family enzymes important for detoxification; expressed in many cancers; hypermethylation of gene promotor found in PCa and several cancers.

*HIF1A*: encodes hypoxia inducible factor 1 subunit alpha, a main regulator of homeostatic response to hypoxia and is essential for vascularization, tumor angiogenesis and ischemic disease; a target of cancer therapy; expressed in all cancers and correlated with cancer relapse and metastasis.

*HPN*: encodes hepsin, a type II transmembrane serine protease involved in blood coagulation and cell morphology; expression associated with cancer growth and progression, especially prostate cancer.

*LMTK2*: lemur tyrosine kinase 3, a serine/threonine/tyrosine kinase involved in nerve growth factor (NGF)-TrkA signalling and endosomal membrane trafficking; expressed in most cancers; found to be a negative regulator of AR activity.

*MYO6*: encodes myosin VI, a motor protein that moves to the minus end of actin filaments and is involved in intracellular transport and maintenance of the ear hair cell structure; expressed in most cancers with high level in prostate cancer.

*PCA3*: produces a spliced, long non-coding RNA; overexpressed in prostate cancer; used as a diagnostic marker to determine the necessity of repeat biopsy.

*PIP5K1A*: encodes phosphatidylinositol-4-phosphate 5-kinase type 1 alpha, a kinase that catalyzes the phosphorylation of phosphatidylinositol 4-phosphate (PI4P) to form phosphatidylinositol 4,5-bisphosphate (PIP2), which is important for the PI3K/Akt/PTEN cancer pathway; involved in actin cytoskeleton organization, cell adhesion, migration and phagocytosis; expressed in many cancers; differentially expressed in metastatic prostate cancer.

*PMP22*: encodes peripheral myelin protein 22, an integral membrane protein and main component of myelin in the peripheral nervous system; mutations caused Charcot-Marie-Tooth, Dejerine-Sottas syndrome, and hereditary neuropathy; expressed in many cancers.

*KLK3*: encodes kallikrein related peptidase 3, member of a subgroup of serine proteases with diverse physiological functions and involved in carcinogenesis; serum level of the protein used in the diagnosis and monitoring of prostatic cancer.

*PSCA*: encodes prostate stem cell antigen, a glycosylphosphatidylinositol-anchored cell membrane glycoprotein; expressed in prostate, bladder, placenta, colon, kidney, and stomach; up-regulated in prostate, bladder and pancreatic cancers.

*PTEN*: encodes phosphatase and tensin homolog, which dephosphorylates phosphoinositide substrates and negatively regulates intracellular levels of phosphatidylinositol-3,4,5-trisphosphate as a tumor suppressor by negatively regulating the PI3K/AKT pathway; mutations found in many [cancers](https://en.wikipedia.org/wiki/Cancer).

# *TMPRSS2*: encodes transmembrane serine protease 2, which is involved in several physiological and pathological processes; expression up-regulated by androgen in prostate cancer and down-regulated in androgen-independent prostate cancer; *TMPRSS2:ERG* fusion found in many prostate cancer patients.

*VEGFA*: encodes vascular endothelial growth factor A, a heparin-binding growth factor, which promotes proliferation and migration of vascular endothelial cells and is important for angiogenesis; up-regulated in many cancers and correlated with cancer stage and progression.

**Table S4** Diagnostic performance of the 25-Gene Panel in GSE17951 prostate tissue specimen cohort (n=154)

|  | Positive | Negative | Total |
| --- | --- | --- | --- |
| PCa | 55 | 0 | 55 |
| Non-PCa | 4 | 95 | 99 |
| Total | 59 | 95 | 154 |
| Sensitivity (95% CI) | 100% (100-100%) | | |
| Specificity (95% CI) | 96.0% (99.8-92.1%) | | |
| PPV (95% CI) | 93.2% (99.6-86.8%) | | |
| NPV (95% CI) | 100% (100-100%) | | |
