# Supplementary material for: Development and validation of a 25-Gene Panel urine test for prostate cancer diagnosis and potential treatment follow-up
Source: BMC Med. 2020 Dec 1;18:376. doi: 10.1186/s12916-020-01834-0 (PMC7706045; doi:10.1186/s12916-020-01834-0)

**Development and Validation of a 25-Gene Panel Urine Test for Prostate Cancer Diagnosis and Potential Treatment Follow-up**

**Additional File 3**

**Supplementary Figures**

**Figure S1** Box plots of biomarkers with increased gene expression levels in prostate tissue specimens from patients with prostate cancer as compared to patients with benign prostate in the GSE17951 cohort (n=154)

**
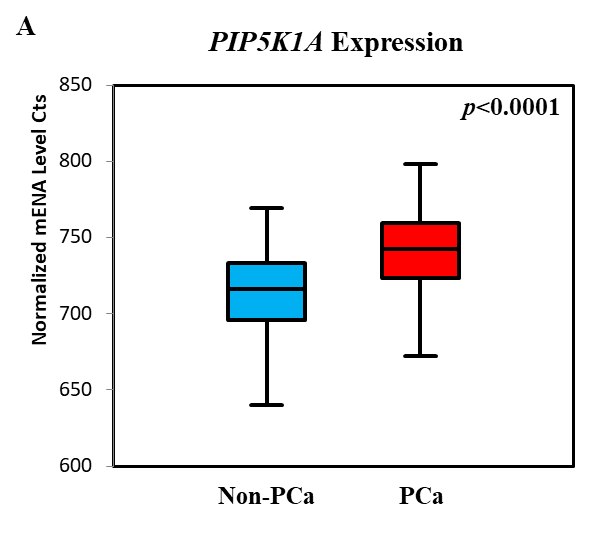

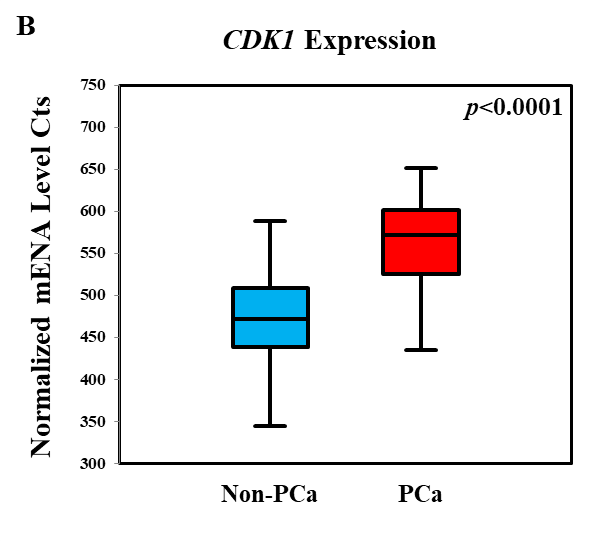
**

**
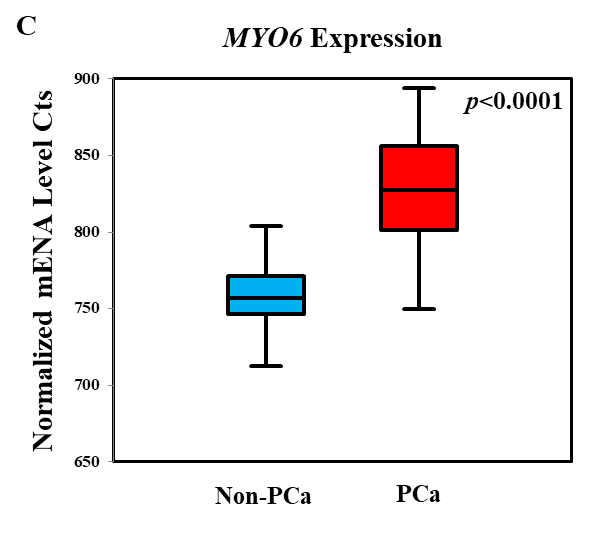

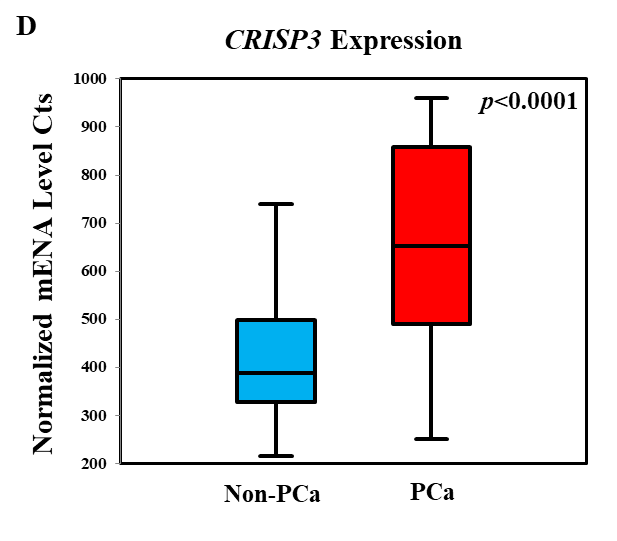
**

**
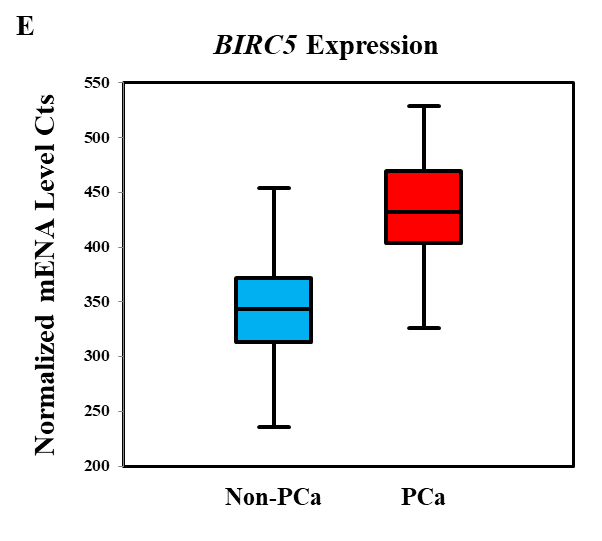

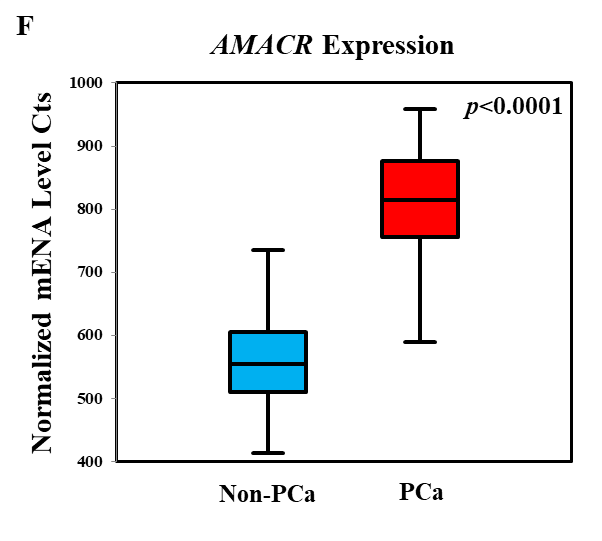
**

**
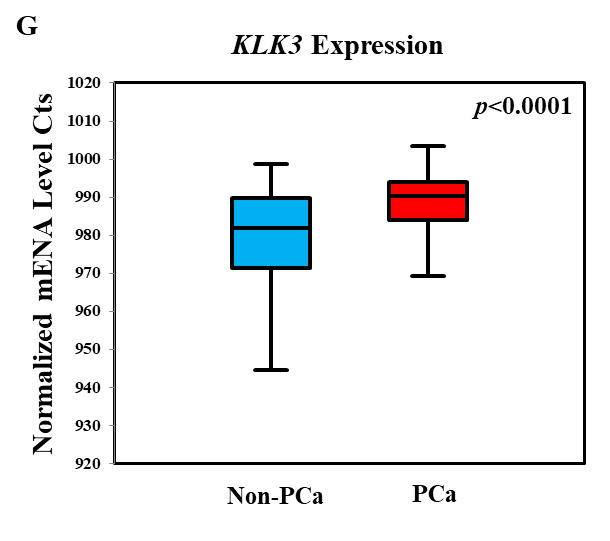

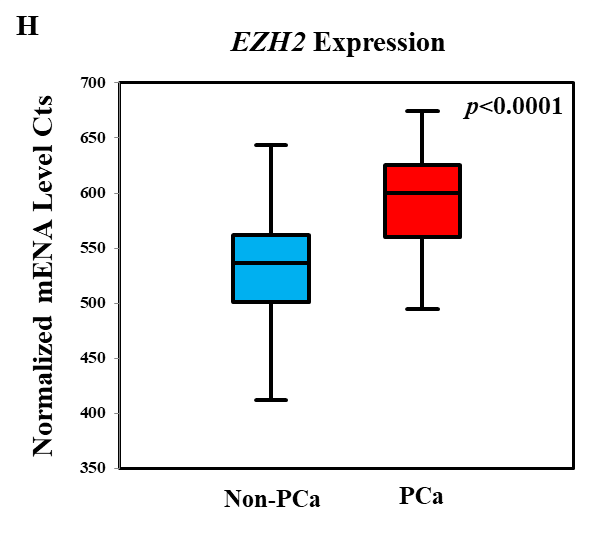
**

**
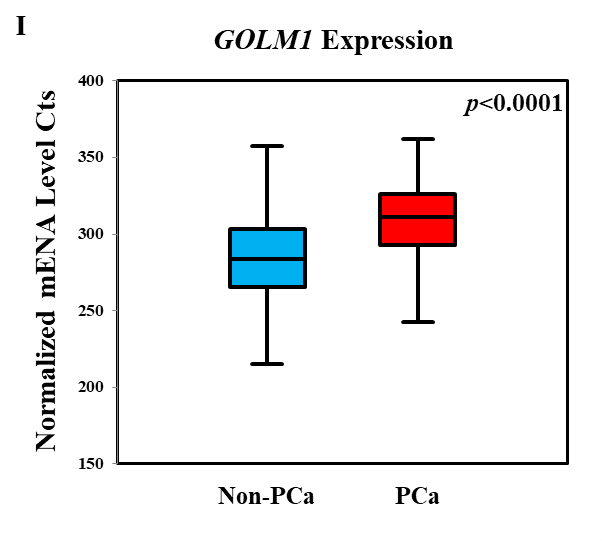

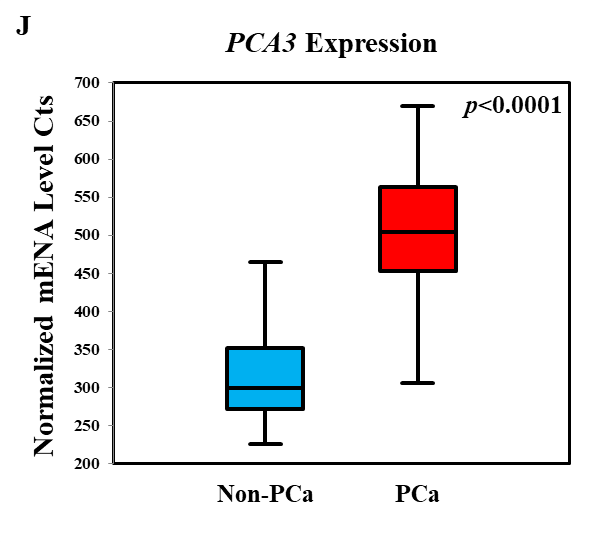
**

**
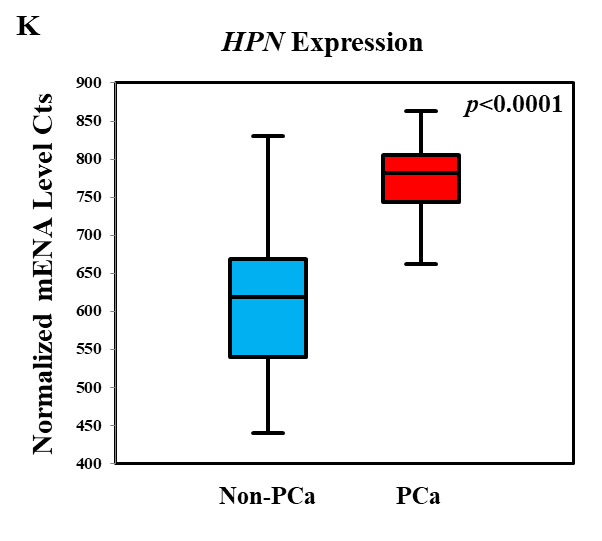

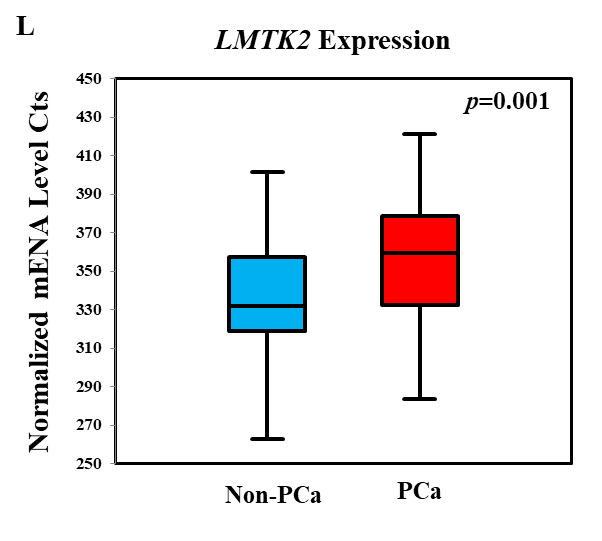
**

**
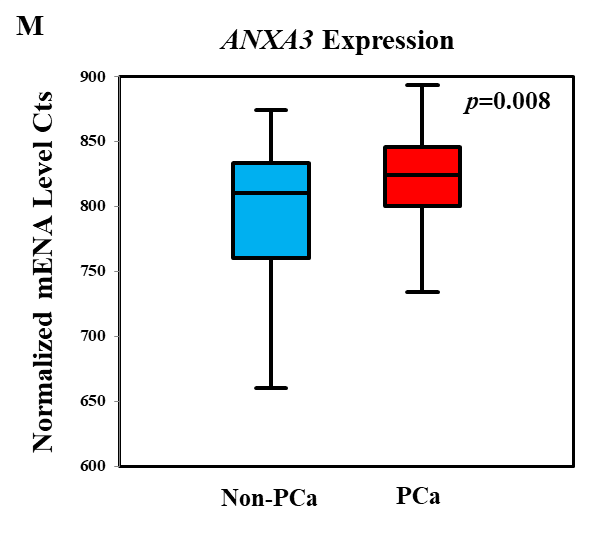

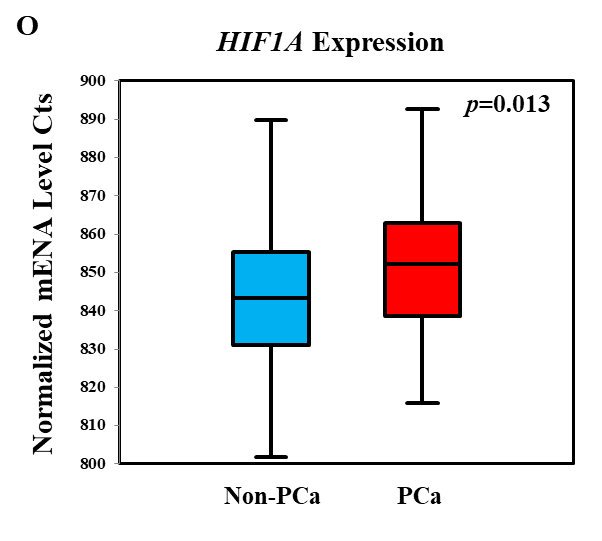
**

**
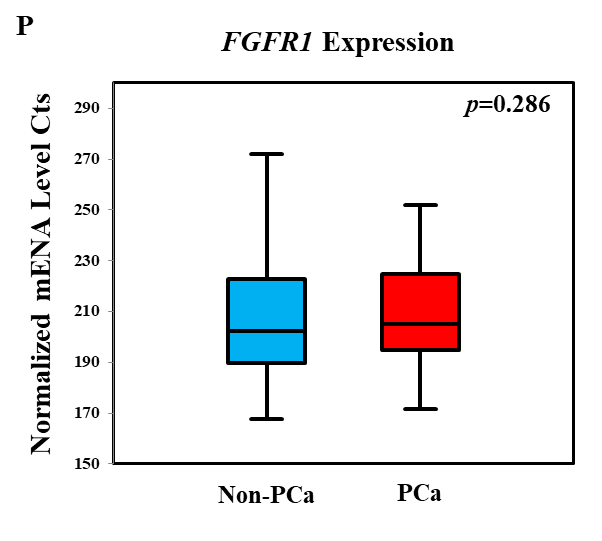

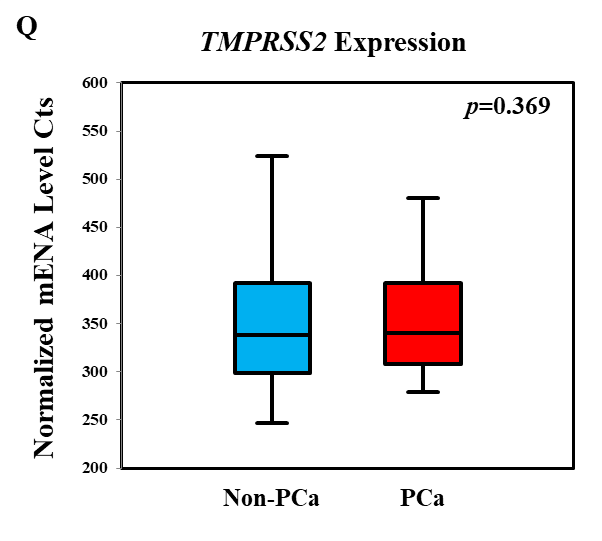
**

**
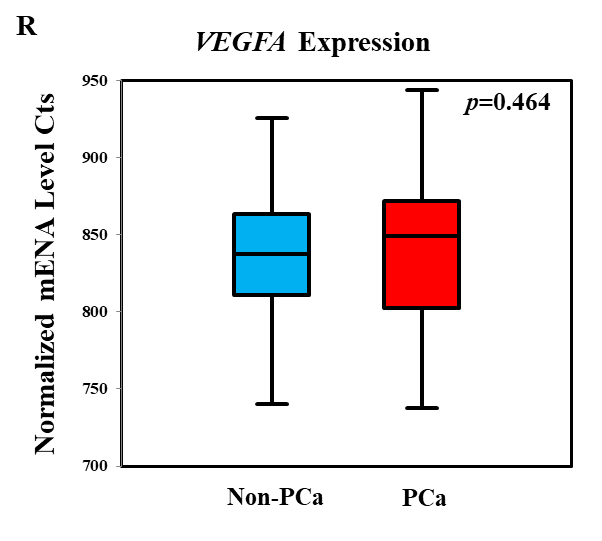

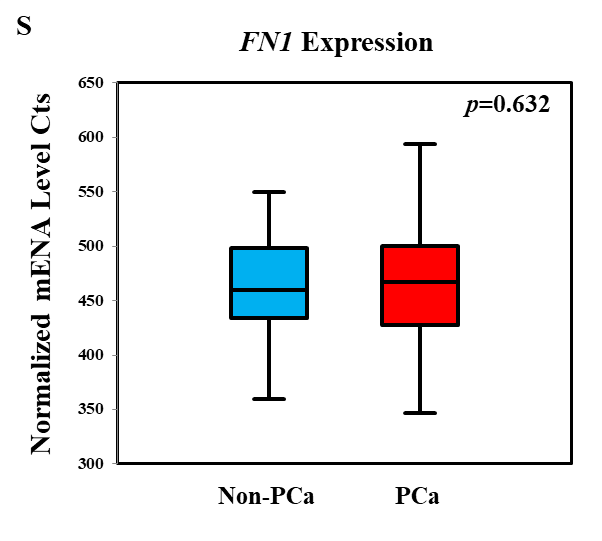
**

**Figure S2** Box plots of biomarkers with decreased gene expression levels in prostate tissue specimens from patients with prostate cancer as compared to patients with benign prostate in the GSE17951 cohort (n=154)

**
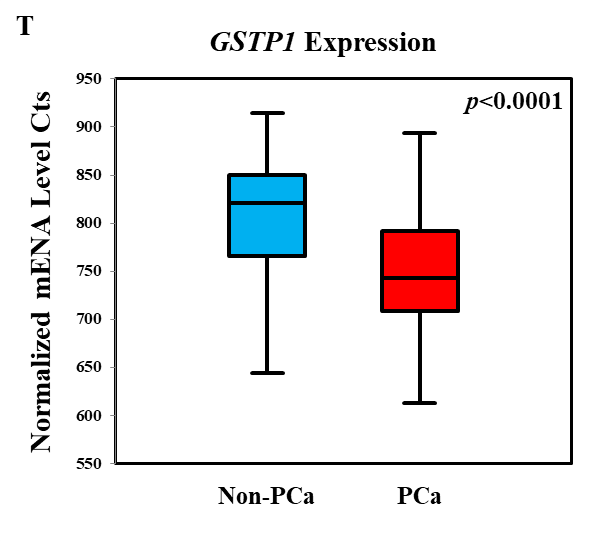

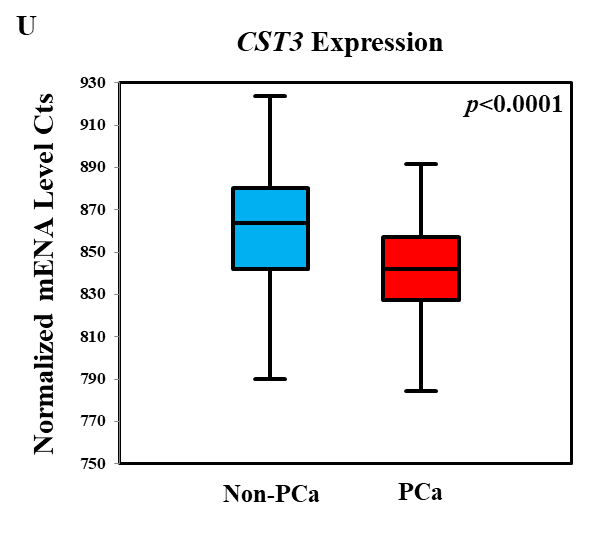
**

**
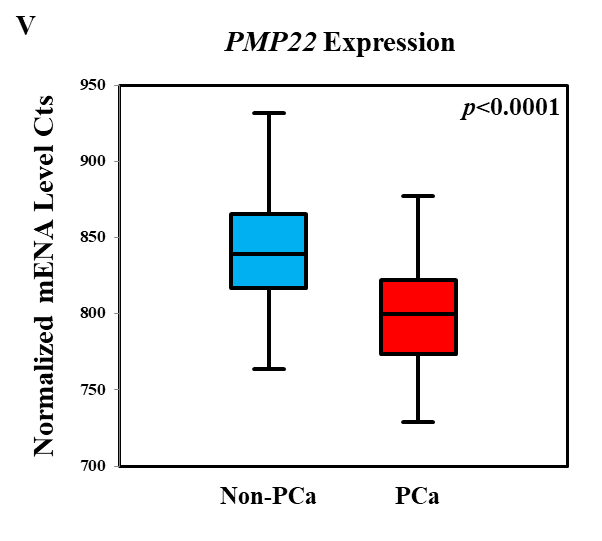

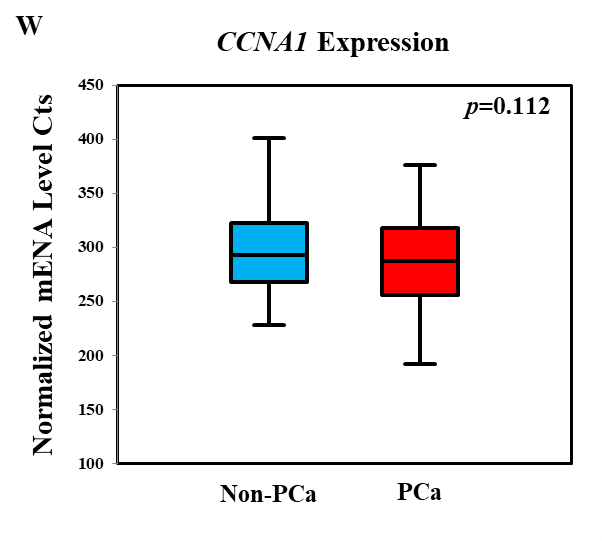
**

**
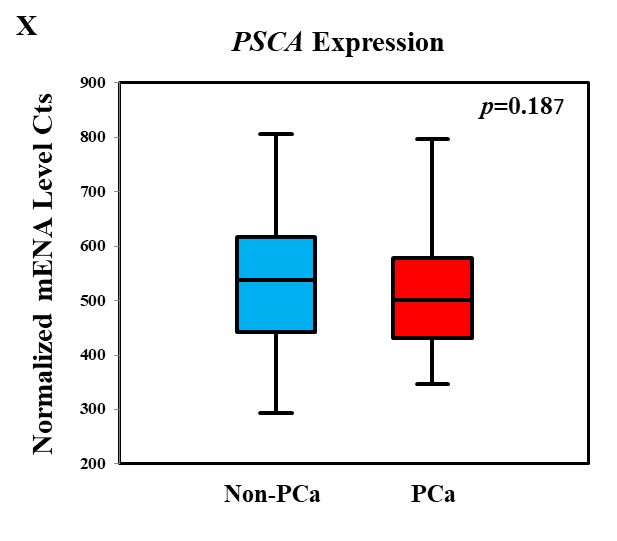

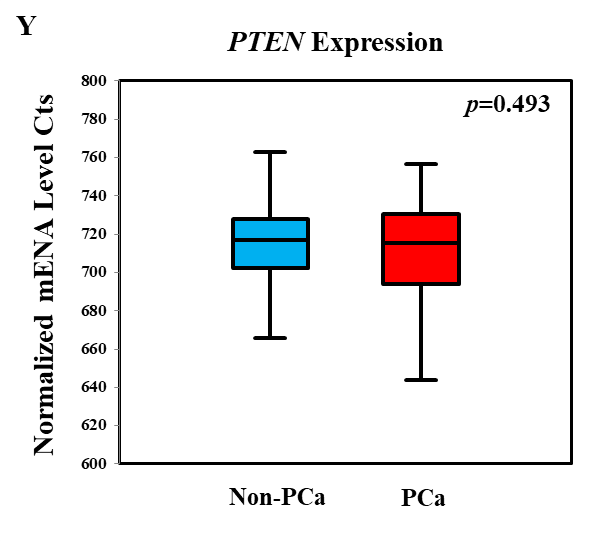
**

**Figure S3** Box plot of discriminant score F1 of the 25-Gene Panel in prostate tissue specimens from patients with prostate cancer as compared to patients with benign prostate in the GSE17951 cohort (n=154)


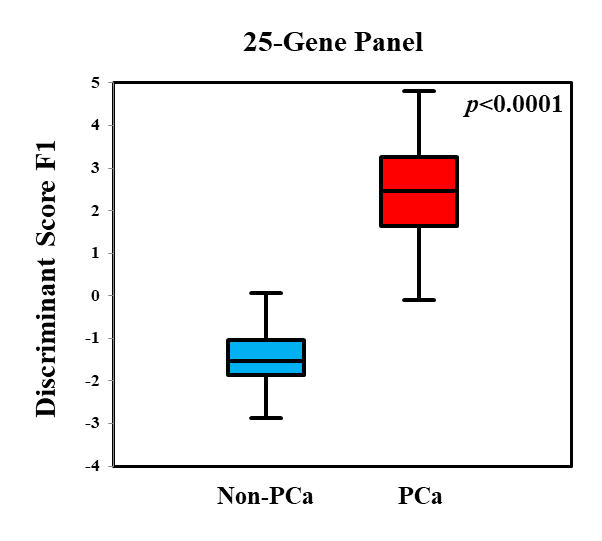


**Figure S4** Receiver operating characteristic (ROC) curve of the 25-Gene Panel for PCa diagnosis in GSE17951 prostate tissue specimen cohort (n=154)


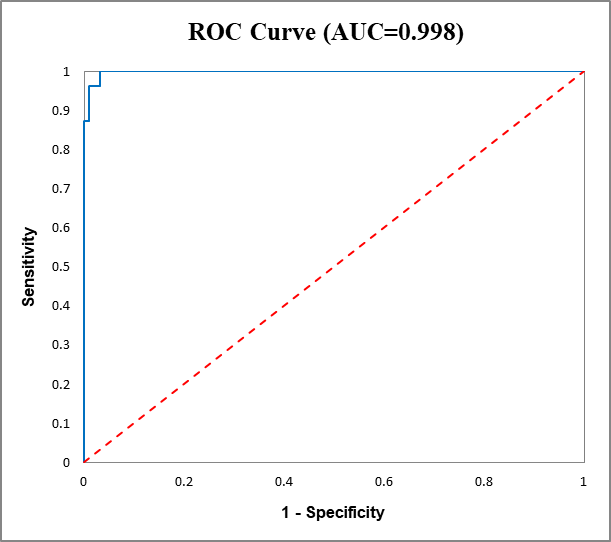

Supplement: Supplementary file 3 — Additional file 3. Supplementary figures, including box plots of biomarkers with increased gene expression levels in prostate tissue specimens from patients with prostate cancer as compared to patients with benign prostate in the GSE17951 cohort (n = 154) (Fig. S1), box plots of biomarkers with decreased gene expression levels in prostate tissue specimens from patients with prostate cancer as compared to patients with benign prostate in the GSE17951 cohort (n = 154) (Fig. S2), box plot of discriminant score F1 of the 25-Gene Panel in prostate tissue specimens from patients with prostate cancer as compared to patients with benign prostate in the GSE17951 cohort (n = 154) (Fig. S3), and receiver operating characteristic (ROC) curve of the 25-Gene Panel for PCa diagnosis in GSE17951 prostate tissue specimen cohort (n = 154) (Fig. S4). [file 12916_2020_1834_MOESM3_ESM.docx]
